# Neonatal oral myiasis caused by the larvae of *Sarcophaga ruficornis* (Diptera: Sarcophagidae): a case report

**DOI:** 10.1186/s12879-021-06742-z

**Published:** 2021-10-15

**Authors:** Minyu Zhou, Ke Cao, Hui Huang, Xiaojuan Luo, Ying Wang, Weike Ma, Zhiyue Lv

**Affiliations:** 1 Key Laboratory of Tropical Disease Control (Sun Yat-Sen University), Ministry of Education, Guangzhou, China; 2 Key Laboratory of Tropical Translational Medicine of Ministry of Education, Hainan Medical University, Haikou, China; 3 NHC Key Laboratory of Control of Tropical Diseases, Hainan Medical University, Haikou, China; 4 Shenzhen Children’s Hospital, Shenzhen, China

**Keywords:** Neonatal oral myiasis, Nosocomial, Molecular identification

## Abstract

**Background:**

Myiasis is caused by dipterous larvae, and rarely affects the mouth. Diagnosis by traditional means is easy to be confused with other similar species. Here, we report a case of oral myiasis, in a 5-month-old infant who was diagnosed by morphological examination and molecular biological methods.

**Case presentation:**

A 5-month old infant with acute myeloid leukemia was admitted due to recurrent skin masses for more than 4 months. The infant had lip swelling, which prevented him from closing the mouth and membranes were present in his mouth and there were also oral ulcers and erosions. Ten maggots were found in the mouth and one in the ear canal with pus flowing out and were confirmed as the third stage larvae of *Sarcophaga ruficornis* by morphological examination and a comparison of sequence of cytochrome oxidase subunit 1 (*COX1*) gene. After removal of the maggots and chemotherapy, the infant ’s condition was gradually improved.

**Conclusions:**

To the best of our our knowledge, this is the first neonatal oral myiasis case reported in China and its diagnosis requires a high index of suspicion. Microscopy combined with specific DNA sequence analysis is an effective technological tool to provide rapid diagnoses of the larva specimen and cases of rare diseases, as illustrated in the current case.

**Supplementary Information:**

The online version contains supplementary material available at 10.1186/s12879-021-06742-z.

## Background

Myiasis is an infestation in living mammals caused by dipterous larvae [1, 2]. The female flies land on the hosts and deposit eggs and maggots, and the larvae then penetrate the tissues [3]. In humans, myiasis rarely occurs in the mouth [4, 5]. In recent years, many cases of neonatal myiasis have been reported globally, especially in developing countries, with a myriad of symptoms, such as dyspnea, hyperemic inflammation of the tympanic membrane, and even cardiac arrest in severe cases [7–9].

Here, we report a case of oral myiasis, in a 5-month-old infant who was diagnosed by morphological examination, biology techniques and bioinformatics.

### Case presentation

A 5-month-old male infant was referred to Shenzhen Children’s Hospital on July 15, 2019 with recurrent skin swellings for more than 4 months and juvenile cells in the peripheral blood of the patient were detected 24 h ago. His birth history, feeding history, growth history, vaccination history and family history are normal.

The patient was diagnosed as acute myeloid leukemia (AML, M5) based on the clinical examination of blood routine and bone marrow cytology on July 18, 2019. Physician examination at admission showed numerous hard magenta nodules with an indistinct boundary and poor mobility in the head and neck, torso, and limbs, with a maximum diameter of about 6 cm. Skin mass pathology revealed juvenile yellow granulomas. His lips were swollen and not ruddy, especially the upper lip. The lips could not be closed, and he breathed through his mouth. There were numerous white membranes in his mouth. Three days after admission, oral mucosa erosion and oral ulcers appeared, and then 10 maggots were found in the mouth, each about 3 to 5 mm in length, as shown in an additional video file [see Additional file 1]. Meanwhile, a wriggling larva was found in his right ear canal with an outflow of pus. All of them were removed by using sterile tweezers. One of the larvae collected from the patient’s mouth was sent to the Diagnostic Section in the Department of Parasitology of Zhongshan School of Medicine, Sun Yat-sen University.

**Fig. 1:**
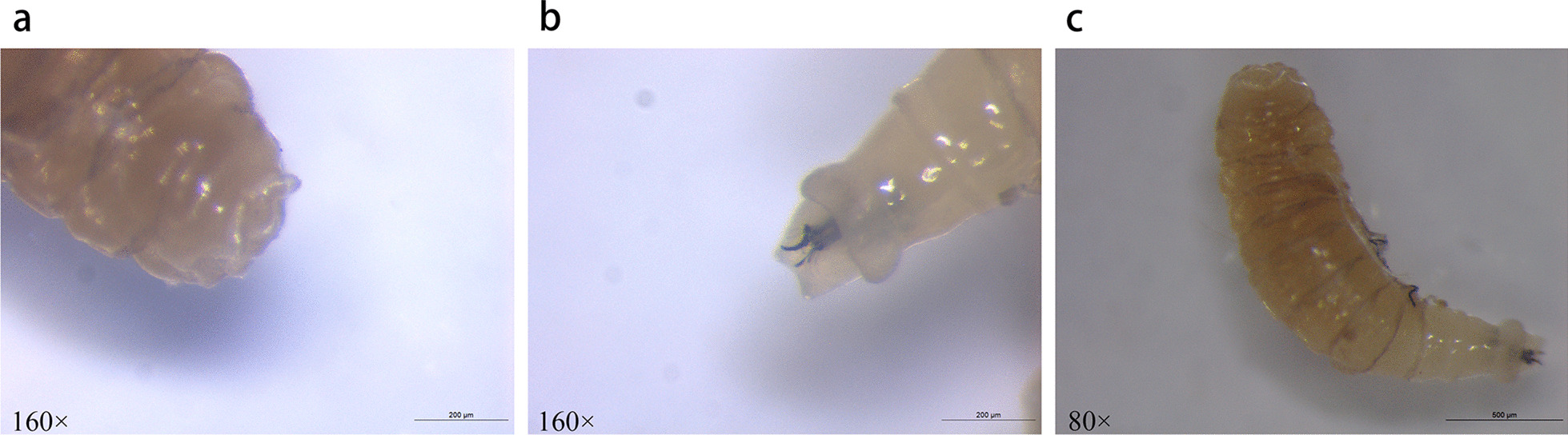
View of the maggot under a stereoscopic microscope. **a** Appearance of the posterior spiracles. **b** Cephaloskeleton and anterior stigmas. **c** Appearance of the whole maggot

**Fig. 2:**
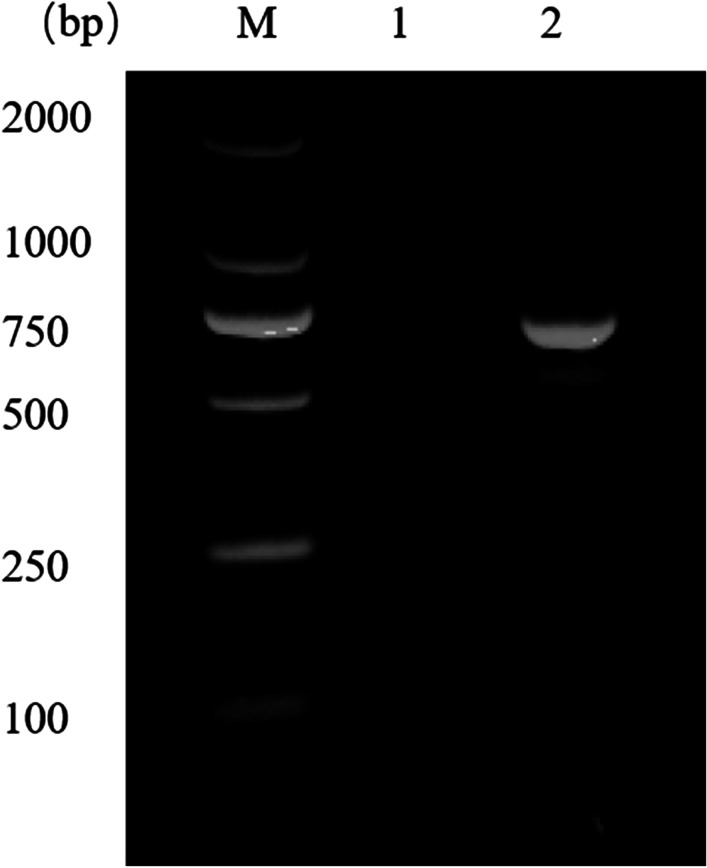
Electrophoresis of the PCR-amplified the cytochrome oxidase subunit 1 (*COX1*) gene products from larva DNA. Lane M: DL2000 DNA marker; Lane 1: PCR product without DNA; Lane 2: *COX1* PCR product. M, Marker; DL, DNA Ladder; COX1, cytochrome oxidase subunit 1; bp, base-pairs

**Fig. 3:**
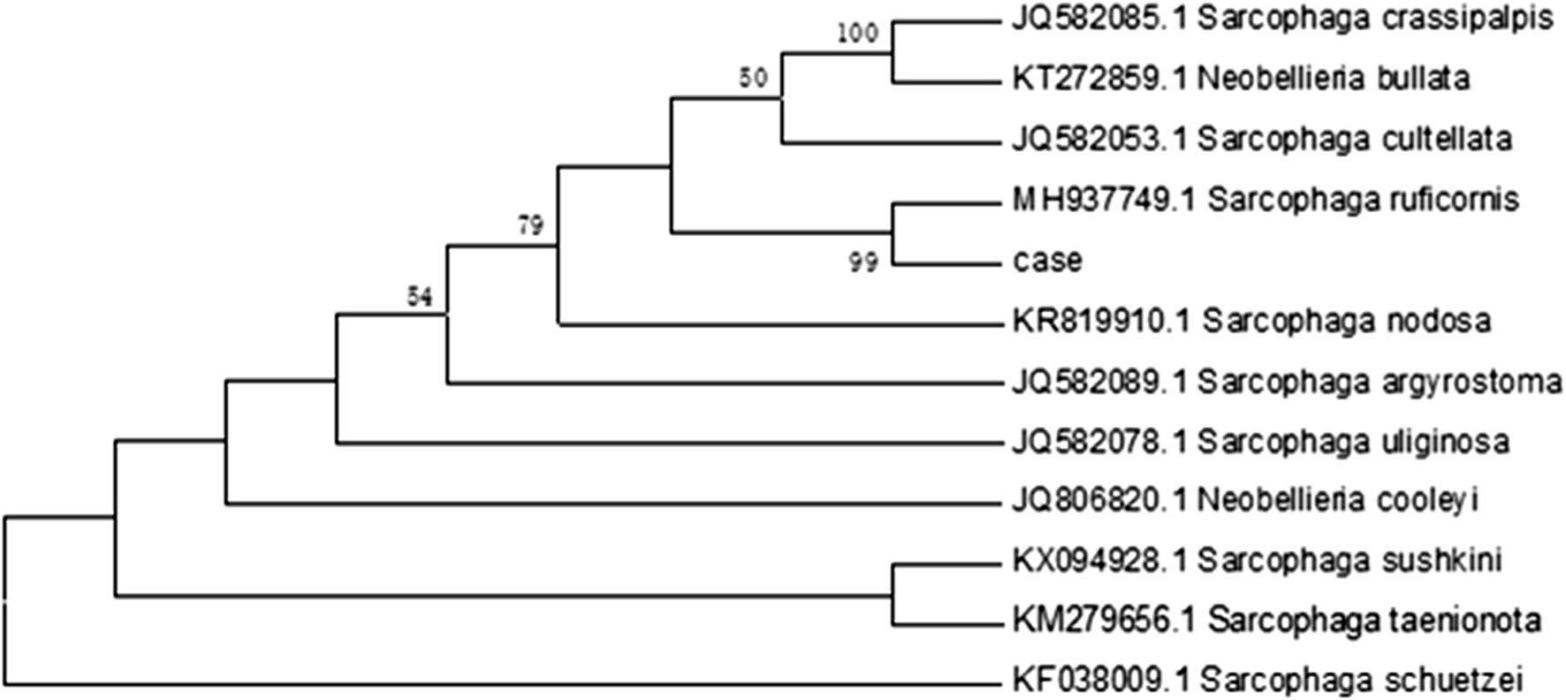
The maximum likelihood tree based on the *COX1* sequences obtained in this study and other related sequences using MEGA 6.0. COX1, cytochrome oxidase subunit 1

The larva (1 mg) was white and about 5 mm in length. The larva collected showed the morphological characteristics of the third instar larva of Brachycera (Fig. 1). Next, we extracted total DNA of the larva using a Hipure Insect DNA Kit (Magen, China) and amplified the cytochrome oxidase subunit 1 (*COX1*) gene by Polymerase Chain Reaction (PCR) using dipteran-specific primer pairs: LCO1490, 5’-TAAACTTCAGGGTGACCAAAAAATCA-3’ and HCO2198, 5’-GGTCAACAAATCATAAAGATATTGG-3’ [10] (Fig. 2) followed by sequencing (Tsingke Co.). BLASTn search for homology in the National Center for Biotechnology Information (NCBI) database (http://blast.ncbi.nlm.nih.gov/Blast.cgi) showed that the sequence had 100% homology with the *COX1* gene of *Sarcophaga ruficornis*. We constructed phylogenetic trees by MEGA 6.0 software, based on 1000 bootstrap replicates from sequences we obtained. Sequences of the homologous genes, with accession numbers of MH937749.1, JQ582053.1, JQ582085.1, KT272859.1, KR819910.1, JQ806820.1, JQ582089.1, KX094928.1, KM279656.1, JQ582078.1 and KF038009.1, of Genus *Sarcophaga* and Genus *Neobellieria* were downloaded from NCBI. The results of the similarity analyses on the basis of the above sequences revealed that the maggots were accurately identified as *S. ruficornis* with 100% sequence matching identities (Fig. 3).

After removal of the maggots in the mouth and strengthening oral care, the oral health of the patients improved and no maggot infection recurred. Moreover, the patient received subsequent chemotherapy with the DAE regimen (cytarabine, daunorubicin, and VP-16), anti-infection and ventilator-assisted respiratory therapy, continuous renal replacement therapy (CRRT) support therapy, and correction of coagulation function. The patient gradually improved following 2 months of symptomatic treatment, and was discharged on September 24, 2019.

### Discussion and conclusion

This is the first neonatal oral myiasis case reported in China. Oral myiasis is rare because the mouth cannot provide a conducive habitat and oral tissues are not directly exposed to the external environment [11, 12].

In this case, the infant could not close his mouth to breathe because of lip swelling and oral ulcers developed three days after admission. His immunity had been compromised by his AML and related treatment. Nocturnal mouth breathing and oral carcinoma are predisposing factors of oral myiasis [13], and the larvae may invade the necrotic tissues through wound. The infant was too young to articulate foreign body sensation or pain in the mouth, let alone the history of being disturbed by flies. Additionally, the summer is the peak season for flies owing to the warm and humid environment [14] and infants are more susceptible to being infected because they have no ability to defend against the flies. Thus, we presume that this is nosocomial according to the patient’s symptoms. Hospitals should pay more attention to sanitary conditions and take certain measures to control mosquitoes and flies, such as using screens and mosquito nets [15]. Emphasizing the oral hygiene of patients, especially infants, is also essential for prevention of myiasis.

We confirmed a myiasis caused by *S. ruficornis* in this case. As a result, clinicians could not have a deep understanding of this disease. Due to lack of personnel in parasite identification, relatively subjective morphological results, and clinicians’ lack of experience in myiasis, simple morphological examination may easily lead to missed or inaccurate diagnosis. Fortunately, more and more methods are being developed to diagnose myiasis. Recently, molecular biology techniques have been popular in diagnosing myiasis, and sequences analysis and phylogenetic analysis have been particularly widely applied [16–20]. Dermoscopy [21], ultrasound scanning [22], mineral oil and magnification [2], serology [23] and many other techniques are applied to diagnose myiasis. Because molecular biological methods can avoid misjudgment and lead to specific detection compared to the traditional methods, they increase accuracy and efficiency of species determination and diagnosis. Moreover, they can help to understand the development of species and explore the relationships between species in order to obtain better prevention and treatment. In summary, we combined morphological observation and molecular investigation to identify *S. ruficornis* as the cause of oral myiasis.

## Supplementary Information

**Additional file 1.** The maggots removed from the patient’s mouth. After admission for 3 days, live maggots about 3–5 cm long appeared in the patient’s mouth, and they were removed.

## Data Availability

All data generated or analysed during this study are included in this published paper.

## Abbreviations

AML: Acute myeloid leukemia
CRRT: Continuous renal replacement therapy
PCR: Polymerase chain reaction
COX1: Cytochrome oxidase subunit 1
